# Applications of Romanian Propolis in Phyto-Inhibitory Activity and Antimicrobial Protection: A Comparative Study

**DOI:** 10.3390/antibiotics12121682

**Published:** 2023-11-30

**Authors:** Ramona Cristina Heghedűş-Mîndru, Mirel Glevitzky, Gabriel Heghedűş-Mîndru, Gabriela-Alina Dumitrel, Maria Popa, Doriana Maria Popa, Isidora Radulov, Mihaela Laura Vică

**Affiliations:** 1 Faculty of Food Engineering, University of Life Science “King Mihai I”, 300645 Timișoara, Romania; ramonaheghedus@usab-tm.ro (R.C.H.-M.); gabrielheghedus@usab-tm.ro (G.H.-M.); isidoraradulov@yahoo.com (I.R.); 2 Faculty of Exact Science and Engineering, “1 Decembrie 1918” University of Alba Iulia, 510009 Alba Iulia, Romania; mpopa@uab.ro; 3 Faculty of Industrial Chemistry and Environmental Engineering, Politehnica University of Timisoara, 300223 Timișoara, Romania; alina.dumitrel@upt.ro; 4 Clinical Emergency Hospital for Children Cluj Napoca, 400398 Cluj-Napoca, Romania; popa.dorianaa@yahoo.com; 5 Department of Cellular and Molecular Biology, “Iuliu Hațieganu” University of Medicine and Pharmacy, 400012 Cluj-Napoca, Romania; mvica@umfcluj.ro

**Keywords:** chemical analysis, phyto-inhibitory activity, antimicrobial activity, propolis, cereals

## Abstract

Propolis use in medicine, pharmaceutical, cosmetic, and food industries is well known. This study aimed to investigate propolis’ phyto-inhibitory and antimicrobial potential. Nine propolis samples obtained from distinct Romanian regions and characterized in terms of physical–chemical parameters, phenols and flavonoid contents, and antioxidant properties were prepared as dry propolis and aqueous extracts. The phyto-inhibitory effect was comparatively tested on different cereals: hexaploid bread wheat (*Triticum aestivum*), maize (*Zea mays* L.), oats (*Avena sativa* L.), and barley (*Hordeum vulgare* L.), while their in vitro antimicrobial activity was evaluated against bacterial and fungal strains specific to cereals: *Bacillus subtilis*, *B. cereus*, *Proteus mirabilis*, *Fusarium oxysporum*, *Penicillium chrysogenum*, and *Aspergillus niger*. All propolis samples showed a phyto-inhibitory effect on the cereals, the most pronounced being corn and oats. Propolis powder samples displayed a lower phyto-inhibitory activity than propolis extracts. Also, all tested products showed inhibitory efficacy against both bacteria and fungi. Furthermore, principal component analysis showed differences between the samples’ phyto-inhibitory and antimicrobial properties depending on the geographical origin. Positive correlations were found between the polyphenols, flavonoid content, and antioxidant activity, respectively. These data support propolis’ phyto-pharmaceutical potential related to its use in plant crop management as an alternative in ecological agriculture.

## 1. Introduction

Bees are important to agriculture and the environment because they contribute to plant reproduction through pollination [1]. The products obtained from bees, besides honey, are propolis, pollen, royal jelly, and beeswax [2]. Propolis is a natural aromatic, resinous substance produced by bees to cover the cracks in the inner walls of the hive and other living things that enter it [3]. It has bactericidal therapeutic properties [4], antiviral [5], antifungal [6], anti-inflammatory, anesthetic, analgesic, regenerative, and antitoxic [7], among other biological activities. Propolis also has a strong bioactive action and is a natural medicine used since ancient times. The medicinal properties were known by the Greeks and Romans, and it was used as an antiseptic and cicatrizing agent in the treatment of wounds [8]. Propolis is a resinous vegetable substance that bees create by mixing their own saliva with beeswax, to which they add components derived from plants and trees [9]. More than 500 chemical compounds have been identified in propolis to date: flavonoids, terpenoids, phenolic and fatty acids, vitamins, amino acids, sugars, proteins, and minerals [10,11].

Over time, most research has focused on potentially treating various acute and chronic diseases with propolis produced by bees as alternative complementary medicines [12]. But there are also studies that show that propolis can be used in agriculture, especially in the control of phytopathogens in crops. Moraes et al. [13] studied the use of propolis in tomato crops to control phytopathogens. With the same aim, other studies highlighted the use of propolis in the cultures of coffee [14], beans [15], cucumbers [16], and grapes [17].

Although propolis can be used as a powder in agriculture, it is often necessary for it to be in the form of a concentrated solution of propolis (aqueous, alcoholic extract–tincture). The use of propolis in agriculture is relatively recent, especially due to the promotion of organic agriculture [18]. In the scientific literature, a series of extraction methods (maceration, ultrasound, and microwave applications) in different solvents (ethanol and water) are described [19].

Propolis can protect plants as an alternative that can be applied in crop systems to avoid or reduce pesticide use that harms plants, the environment, and the population [11]. Natural production methods from organic farming encourage the use of propolis in agriculture. If it is widely incorporated in crop management, its bioactive and economic importance will increase in the future because foods produced using propolis are qualitatively different from foods produced using conventional methods. When considering natural products, certain aspects of the sustainability and quality of organic food are highlighted [20]. The bioactive compounds of propolis must be extracted from it so that the final solution can be applied to the crops. Due to the large number of chemicals, some of its compounds are soluble in water or alcohol or both solvents [21].

The multitude of bioactive components of propolis has determined its use in various fields such as medicine, pharmaceuticals, cosmetics, and food industries [22]. Although the antimicrobial effect has been intensively studied, most studies have been conducted on a limited number of strains or strains pathogenic to humans and less on microorganisms affecting plants.

The flavonoids and phenols in propolis help prevent and eliminate microbial and fungal infections that can affect grains, such as molds and pathogenic bacteria [23,24] and can increase plant resistance to abiotic stresses, such as drought, soil salinity, or extreme temperatures. These compounds can help regulate plant physiological processes to better cope with these extreme conditions [25]. So, propolis can have a good effect on grains, providing protection against pathogens, helping them cope with difficult environmental conditions, and contributing to good growth.

Additional research is required for the development of commercial products based on propolis–ethanolic and aqueous extracts at different concentrations with applicability in agriculture. The present study highlights the physico–chemical parameters of propolis collected from the regions of Romania and the effect of propolis powder or aqueous extracts on different crops. At the same time, the antimicrobial effect of propolis on some strains of microorganisms specific to cereals is presented.

## 2. Results and Statistical Analysis

### 2.1. Samples Characterization

The values of the physico–chemical parameters for the nine propolis samples collected from different regions of Romania are presented in Table 1.

The values obtained for water activity are between 0.62 and 0.78. Regarding the hygroscopicity, the value for all samples ranges between 12.6 and 14.7 g of absorbed water per 100 g of propolis. The water solubility of propolis samples is between 8.26 and 13.43%. In the case of the total phenolic content of all nine propolis samples, the values ranged from 102.7 (sample 6 from the Muntenia region) to 189.4 mg (sample 7 from the Dobruja region) of GAE/g. In contrast, the total flavonoid content was quantified between 65.30 (sample 6 from the Muntenia region) and 85.19 mg (sample 7 from the Dobruja region) of QE/g. The maximum number of phenols and flavonoid amounts was recorded in samples 1 and 7 from Alba and Constanța County, and the lowest value was in sample 6 from Dâmbovița County. The DPPH and FRAP assays estimate the antioxidant activities of propolis samples collected from all areas of Romania. Samples of propolis showed high antioxidant capacity.

### 2.2. Phyto-Inhibitory Activity of Propolis Samples

In Supplementary Tables S1–S8, the plume lengths for 13 days (growth rate) are presented comparatively for wheat, corn, barley, and oat samples, over which different quantities of powdered propolis were applied (1, 5, and 10 g), and aqueous extracts of propolis at different concentrations (1%, 5%, and 10%). The samples were collected from the nine regions of Romania. The control sample (M) is the one for which propolis was not applied.

In the case of the average plumule growth lengths for the wheat samples (Supplementary Table S1), it is found that the slowing down of plant growth is directly proportional to the amount of propolis applied over time. After 13 days, for the doses of 1 g and 10 g, samples S4 from Timiș County and S9 from Suceava had the lowest growth. Also, when 5 g of propolis is applied, sample S4 records the lowest plume growth at the end of the monitoring period. In the wheat samples, the radicle and the plumule are visible even from the first days, except for the samples in which 10 g of propolis was applied. The control sample, without propolis inhibitor, has the fastest growth.

The wheat samples treated with different concentrations of aqueous propolis extracts (Supplementary Table S2) have a similar tendency to inhibit grain growth as those treated with propolis powder. In the case of wheat samples, the plume growth speeds are relatively close for all nine propolis samples. Samples S4 and S9 show the most pronounced inhibitory effect. Plumule growth lengths are comparatively smaller when using concentrations of aqueous propolis extracts of 1%, compared to the 10% solution.

From the results obtained, it can be seen that the phyto-inhibitory activity is higher when propolis powder is applied than when aqueous solutions are used.

As with wheat, the plume growth trend is similar in the case of corn samples treated with propolis powder (Supplementary Table S3). However, the smallest increases in the plume are recorded in the case of samples S7 (Constanța County) and S9 (Suceava County) for the samples to which 1 g of propolis was applied, respectively, S8 (Vaslui County) and S9 (Suceava County) to which was applied a 10 g propolis powder. In the case of corn samples, the plume growth values are similar in the case of dosing 1 g and 5 g of propolis powder as an inhibitor, the difference compared to the control sample being insignificant for all nine propolis samples. The plume growth rate is significantly reduced when 10 g of propolis powder is dosed into corn samples. There is a similarity between the samples to which powder propolis was applied and the case of using aqueous solutions (Supplementary Table S4) of the same inhibitor. However, it is obvious that the propolis solution applied to corn samples has a much stronger inhibitory effect than the powder one.

Among the propolis samples collected from different regions of Romania, there were no large variations in the growth speed of the barley seed plume for doses of 1 g and 5 g. These are more obvious when applying the dose of 10 g, where the maximum difference is 11 mm between sample S4 (Timiș County) with the lowest growth and sample S2 (Bihor County) with the highest growth (Supplementary Table S5). In the case of barley samples, plumule growth lengths are not significantly reduced when treated with inhibitor solutions of different concentrations: 1%, 5%, and 10% (Supplementary Table S6). Overall, plume growth rates are slightly below the control sample (M).

In the case of oat samples (Supplementary Tables S7 and S8), when applying 10 g inhibitory propolis, after 13 days, samples S3 (Maramureș County) and S4 (Timiș County) have the lowest average increase in plumule lengths. In the case of oats, a similar evolution of the plume lengths is constant as in the case of the other cereals but with growth values similar to those of the corn samples. In general, all propolis samples studied inhibited germination significantly compared to the control sample.

### 2.3. Antimicrobial Activity of Propolis Samples

The antimicrobial effect of the nine aqueous extracts of propolis collected from all regions of Romania against six microbial (bacterial and fungal) strains specific to cereals are presented in Table 2.

As shown in Table 2, all propolis extracts showed antibacterial activity against all strains tested, with the diameters of the inhibition zones varying between 16 and 29 mm. Sample S1 (from Transylvania) had the strongest inhibitory effect, and the most sensitive strain was that of *B. cereus*.

The extracts obtained from sample S1 displayed an intense antimicrobial efficacy, with the values of the inhibition zone diameter comparable to those recorded for the positive control (*p* > 0.05).

In the case of bacteria, it can be observed that for *B. subtilis* and *B. cereus,* the diameters of the inhibition zones produced by propolis were smaller than in the case of the antibiotic, but in the case of *P. aeruginosa* sample S1 showed a larger diameter of inhibition than that produced by ciprofloxacin.

All propolis extracts are presented in vitro antifungal activity against *F. oxysporum*, *P. chrysogenum,* and *A. niger.* The diameters of the inhibition zones are smaller than in the case of the antifungal, except for *P. chrysogenum*, where samples S1, S3, and S7 showed larger inhibition zones. 

The comparative effect of Romanian propolis and its aqueous extracts on cereals and the species selected in the research has not been extensively studied.

### 2.4. Analysis of statistical data

#### 2.4.1. Principal Component Analysis

Principal component analysis (PCA) was performed in two steps. In the first stage, the amounts of propolis powder C(1 g), C(5 g), C(10 g), and the control were used as input data (variables). The analysis (PCA) aimed to evaluate the phyto-inhibitory effect of propolis powder, in different amounts, on the four cereal samples, wheat, corn, barley, and oat, after 3, 5, 7, 9, 11, and 13 days.

**Figure 1:**
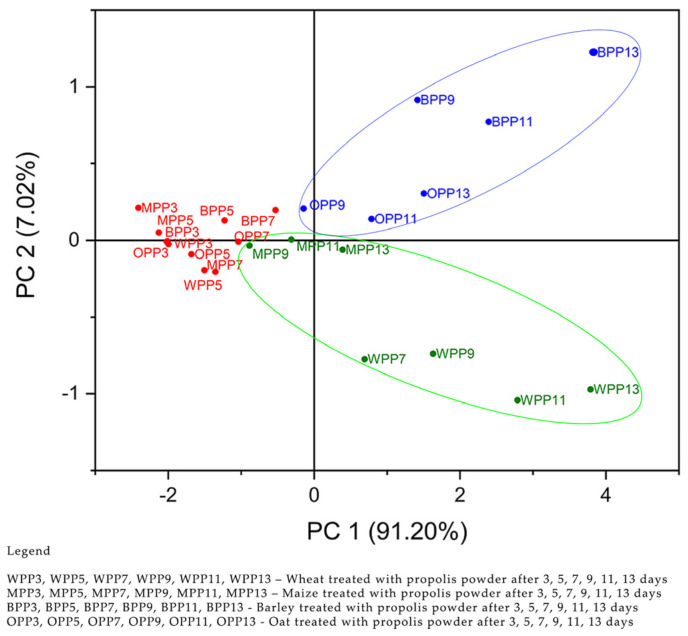
Scores plot of PCA scores of the four cereal samples according to their responses to propolis powder C(1 g), C(5 g), C(10 g), and the control sample.

**Figure 2:**
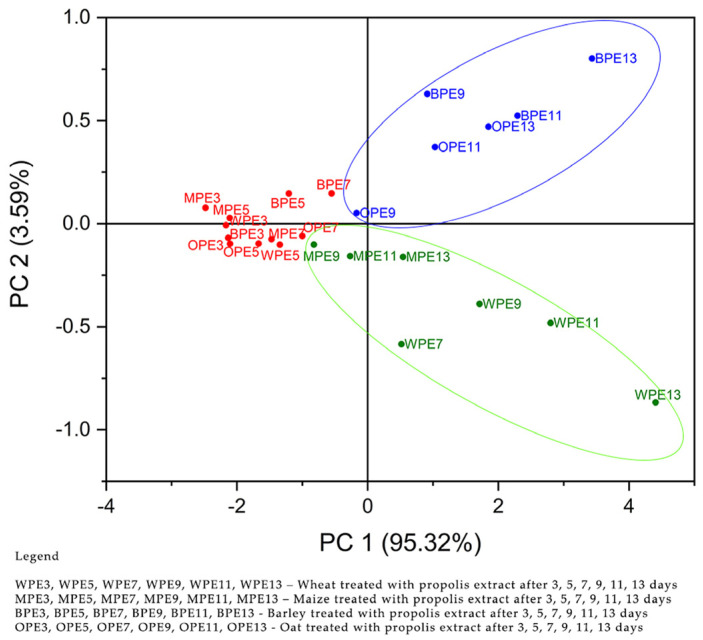
Scores plot for PCA analysis of the four cereal samples according to their responses to propolis extract C(1%), C(5%), C(10%), and the control sample.

Principal components were obtained based on the values of the correlation matrix. The eigenvalues were 3.64, 0.28, 0.057, and 0.013 for PC1–PC4. Figure 1 shows that the first two PCs explain 98.23% of the total data variance, PC1—91.20% and PC2—7.02%. In the second step, concentrations of propolis extracts, C(1%), C(5%), C(10%), and control were used as input data (variables). The purpose of the analysis (PCA) of this paper was to evaluate the phyto-inhibitory effect of propolis extracts of different concentrations on the four samples of cereals, wheat, corn, barley, and oats after 3, 5, 7, 9, 11, and 13 days. The eigenvalues were 3.81, 0.14, 0.035, and 0.0077 for PC1–PC4. In Figure 2, it can be seen that the first two PCs explain 98.91% of the total data variance. PC1—95.32 and PC2—3.59%.

Figure 1 shows observations and PCs obtained from the analyzed data. The first step is the use of propolis powder in the treatment of cereal samples. The scores graph of PC1 versus PC2 shows the formation of two groups of cereal samples. The first group, marked with a blue color and located at the top right, is composed of oat and barley samples after 9, 11, and 13 days of treatment with propolis powder. The second group, marked with a green color and located at the bottom right, is composed of corn and wheat samples after 9, 11, and 13 days of treatment with propolis powder. In the left center of the score graph, marked with a red color, cereal samples that showed a lower response in the first 3 to 7 days to treatment with propolis powder appear.

Figure 2 shows observations and PCs obtained from the analyzed data. Step two shows the use of propolis extract in the treatment of cereal samples. Score plot PC1 versus PC2 shows the formation of the same cereal groups. The first group, marked with a blue color and located at the top right, is composed of oat and barley samples after 9, 11, and 13 days of treatment with propolis extract. The second group, marked with a green color located at the bottom right, is composed of corn samples after 9, 11, and 13 days and wheat after 7, 9, 11, and 13 days of treatment with propolis extract. At this stage, cereal samples, marked with a red color, located in the left central part, treated for 3–7 days with propolis extract did not show a favorable response to the phyto-inhibitory effect, except for the WPE7 sample.

It can be concluded that the values of the variables represented by the amount of propolis powder C 1 g, C 5 g, and C10 g, in the first stage and the concentrations of propolis extracts C1%, C5%, and C10% in the second stage (Figures S1 and S2), are useful in separating grain groups. For the cereal group consisting of oat and barley samples after 9, 11, and 13 days, the variables responsible for the classification were the amount of propolis powder of 10 g and the concentration of 10% propolis extract. For the cereal group consisting of corn and wheat samples after 7, 9, 11, and 13 days, responsible for the classification were the variables represented by the amount of propolis powder of 1 g and 5 g and the concentrations of 1% and 5% propolis solution. Regarding the treatment of cereal samples with propolis extract, it can be stated that it had a greater influence on the inhibition, the WPE7 sample being located in the lower right group of the score graph.

#### 2.4.2. Analysis of Variance (ANOVA)

A two-way ANOVA is used to estimate if the geographical origin of Romanian propolis (nine samples) and the type of strain used (six strains) affect the diameter of the inhibition zone.

Table 3 shows the values obtained related to the dispersion analysis with two inputs for the two-way ANOVA.

Because both in the case of propolis samples and the types of strains F_computed_ > F_0.05_, we will reject the null hypothesis and conclude that the geographical origin of Romanian propolis and the type of strain used have a significant influence on the diameter of the zone of inhibition at a significance level α = 0.05.

#### 2.4.3. Pearson Correlation

A Pearson’s correlation attempts to assess the relationship between the flavonoid and phenol content of propolis samples and the microbial strains used. The Pearson correlation coefficients are presented in Table 4.

There was a statistically significant correlation between the flavonoid content and the diameter of the inhibition zone for the following strains: *B. subtilis* and *P. mirabilis*. A strong correlation is noticed between the phenols content and the diameter of the inhibition zone for the *P. chrysogenum* strain. The correlations between the diameter of the inhibition zone of the strains used and the content of phenols are stronger than in the case of flavonoids.

## 3. Discussion

The results of the physico–chemical properties of propolis from different regions of Romania showed differences between the samples, depending on the geographical origin with its specific flora. The water activity of sample S3 (from the Maramureș region) was 0.62 and 0.78 for S7 (Dobrogea region, Constanța County). According to Sunil et al. [26], samples with high humidity (water content) also show a higher water activity. The Indian crude propolis samples have an a_w_ between 0.68 and 0.73. Brazilian propolis a_w_ was 0.76 for red, 0.8 for green, and 0.87 for brown [27]. Hygroscopicity was relatively low, between 12.6 in S8 from Vaslui County (Moldavia region) and 14.7 g absorbed water/100 g sample in S6 from Dâmbovița County (Muntenia region), and coincides with the research of da Silva et al. [28] where the value is 13.1 g of adsorbed moisture per 100 g of dry solids.

Aqueous extracts of propolis usually contain up to 10 times lower amounts of active ingredients than alcoholic extracts due to the poor solubility of propolis and its bioactive compounds [29]. The water solubility of propolis samples from the regions of Romania varies between 8.26 and 13.43%, having values close to those obtained for Indian propolis, which varies from 8.71 to 19.28% [30].

Propolis has more than 300 natural chemical compounds [31]. Mainly, propolis contains flavonoids, phenolic acids, and their esters. For example, the total phenolic content of Polish propolis ranged from 150.05 to 197.14 mg/g GAE, while the total flavonoid content was 35.64–62.04 mg/g QE [32]. The phenolic content of propolis from Türkiye is 16.73 to 125.83 mg GAE/g sample, while the number of total flavonoids varied from 57.98 to 327.38 mg QE/g sample [33], depending on different geographical origins. The samples taken from all the regions of Romania accumulate large amounts of phenolic compounds (189.4 mg of GAE/g in sample 7 from the Dobruja region), while the highest total flavonoid content was determined for the same sample and is 85.19 mg QE/g.

The antioxidant activity of the propolis was determined using the method with diphenylpicrylhydrazyl (DPPH) and ferric-reducing antioxidant power (FRAP). The results of the two methods used seem to be well correlated [34]. Other research [35,36] indicates that FRAP and DPPH values for propolis analyzed from other countries are compatible with our findings.

The phyto-inhibitory effect of propolis refers to its ability to inhibit seed germination and plant root growth. This effect is due to its high content of bioactive compounds, such as flavonoids and phenolic acids [37], which can interfere with the physiological processes of plants. Although propolis can be used as a natural treatment for weed control in agricultural practice, it must be considered that its use can negatively affect crop plants, depending on the dosage and the application method. For example, doses that are too high may result in plant death, while doses that are too low may have an insignificant effect. The antigerminative activity of propolis has been mentioned in the past [38,39,40], being considered a property attributed to the presence of an antibiotic factor in the composition. Studies targeting the germination percentage and cell division of wheat roots treated with propolis ethanolic extracts significantly inhibited the germination percentage compared to distilled water and control samples [41].

Regarding the effects of propolis on human health, its antioxidant, antimicrobial, anti-inflammatory, and anti-carcinogenic properties have been extensively studied. Studies have shown that flavonoids and other compounds in propolis can help reduce inflammation and protect against cell damage caused by free radicals [42]. Further investigations are needed to understand the exact health effects of propolis and the optimal doses for its use. When using products with propolis for therapeutic purposes, interactions with other drugs or food supplements may occur.

Compared to non-propolis-treated maize grains, the larger grain borer LGB population was lower in propolis-treated grains at all concentrations examined. Propolis’s greatest benefits were observed at a 20% concentration [43].

**Figure 3:**
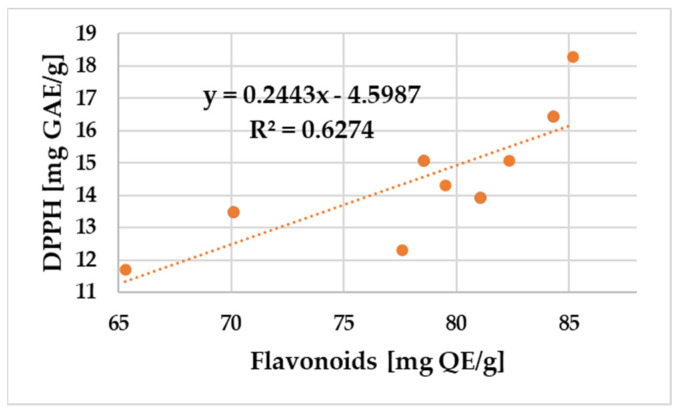
Correlation of total flavonoid content and DPPH free radical scavenging activity of the propolis samples.

**Figure 4:**
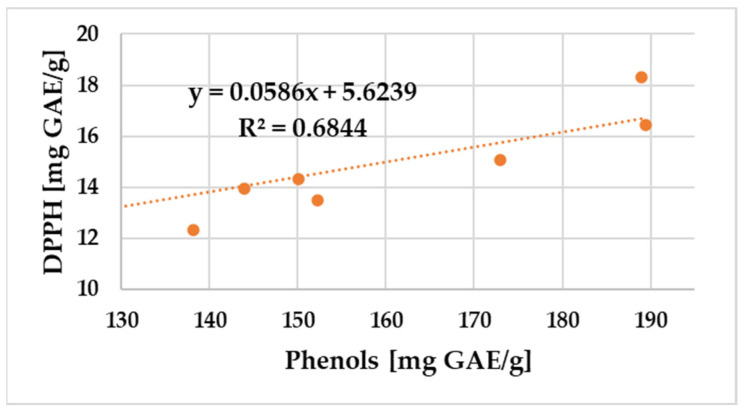
Correlation of total phenolic content and DPPH free radical scavenging activity of the propolis samples.

**Figure 5:**
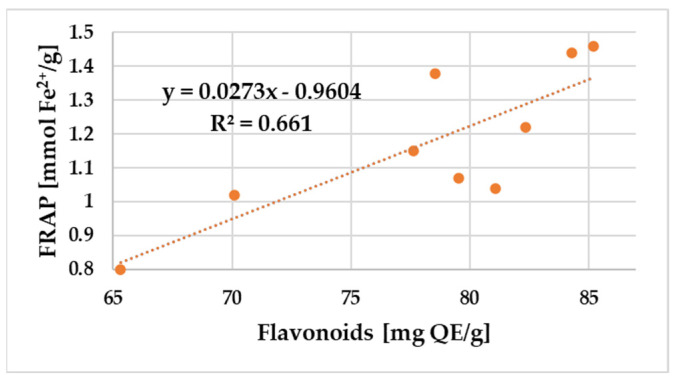
Correlation between total flavonoid content and FRAP in the propolis samples.

**Figure 6:**
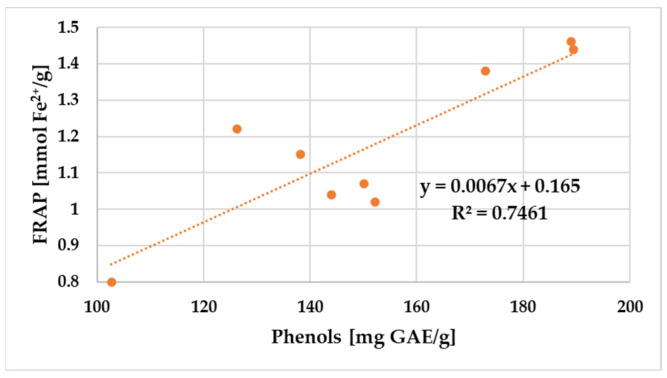
Correlation between total phenolic contents and FRAP in the propolis samples.

A positive correlation was found between the amount of total polyphenols and flavonoid content and the DPPH radical scavenging activity and FRAP values of propolis samples. The relationship between the total phenol and flavonoid content and the antioxidant potential of propolis samples assessed using DPPH and FRAP is shown in Figure 3, Figure 4, Figure 5 and Figure 6.

Figure 5 and Figure 6 show that both total flavonoids (R^2^ = 0.66) and phenolics (R^2^ = 0.75) were moderate and very strongly correlated with FRAP antioxidant activity.

There is not enough scientific evidence to prove that propolis can be used as a natural treatment to combat weeds in agricultural practice. Most of the studies on the properties and benefits of propolis focus on its use in the food, pharmaceutical, or cosmetic industries. However, some research has shown that propolis can inhibit the growth of some species of bacteria and fungi that can affect plants. A study shows that propolis can have an antifungal effect against a fungus that affects potato crops [44,45].

In the case of the nine samples, a significant correlation (R^2^ = 0.63) between the flavonoid content and the free radical scavenging assays was obtained. The total phenolics were also essentially correlated with DPPH antioxidant activity in Figure 4 (R^2^ = 0.68).

In his research, Gonnet [46] demonstrated the phytotoxic and photo-inhibitory properties of propolis applied by bees on potatoes, which exhibited permanent inhibition. In addition, some studies have shown that propolis can have antimicrobial and antifungal properties, making it useful in controlling certain diseases and plant infections. However, these studies were conducted in the laboratory, and more research is needed to evaluate the effectiveness of propolis in agricultural practice.

In general, most of the research on the antimicrobial properties of propolis has been performed in vitro on microorganisms that affect human health, and few studies have focused on the antibacterial or antifungal effect of propolis against species that affect plants used as a food source. For example, a study that tested the antifungal effect of different concentrations of propolis extracts on some species that can affect plants showed that the most affected microorganisms at all propolis concentrations among the tested fungi were *Alternaria alternata* and *Penicillium digitatum* [47]. The results of another study showed that Colombian propolis extracts could act as antifungal agents against *Colletotrichum gloeosporioides* and *Botryodiplodia theobromae*, species that affect food products of vegetable origin [48].

Regarding Romanian propolis, some studies have tried to investigate its antimicrobial activity, confirming its antibacterial effect. Thus, a study on ethanolic extracts of propolis from Transylvania found a better sensitivity of the Gram-positive bacteria (*Staphylococcus aureus*, *Bacillus cereus*, and *Listeria monocytogenes*). At the same time, for *P. aeruginosa,* a total resistance was noticed, and inhibition zone diameters for *Candida albicans* showed a large variation [49]. Another study that investigated the antifungal activity of propolis fractions and ethanolic extracts of propolis from different regions of Romania showed that they exhibited antifungal and antibiofilm activity against all tested *Candida albicans* strains [50]. It was also observed that Romanian propolis ethanolic extracts showed strong antimicrobial efficacy against *E. coli* strains, positively correlated with chemical composition, along with an interesting synergistic interaction with antibiotics [51]. In contrast to these studies that investigated alcoholic propolis extracts, our study used aqueous extracts and demonstrated the antimicrobial activity of propolis.

Socio-economic development in recent years has influenced food contamination with fungi, thus creating the need for new antifungal agents to provide safer food. Our study showed that propolis is one of the promising natural antifungal agents and phyto-inhibitors.

## 4. Materials and Methods

### 4.1. Propolis Samples

Raw brown propolis samples were obtained directly from beekeepers. These were taken from wooden beehives. Sampling was performed by scraping the lid and entering the hives with a stainless steel spatula. The samples were taken during the same period of the year, between June and July 2021, from nine regions of Romania (Transylvania, Banat, Crișana, Maramureș, Oltenia, Muntenia, Dobruja, Moldavia, and Bukovina). The samples were kept at −18 °C in the dark until analysis.

Table 5 shows the number of samples, the region, the county, and the relief form related to where the sample was taken.

**Figure 7:**
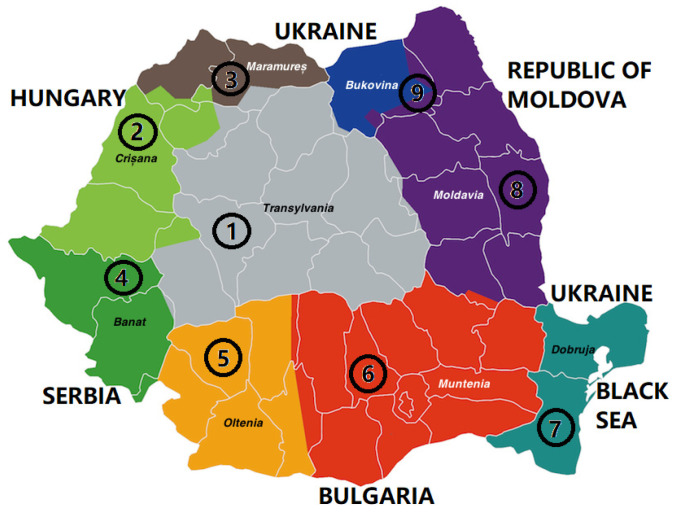
Map of the regions of Romania with propolis sampling points.

Figure 7 shows the map and the regions of Romania, highlighting the counties where the samples were taken.

The numbers from 1 to 9 in Figure 7 represent the areas where the samples from S1 to S9 were taken (Table 5).

### 4.2. Cereal Samples

Table 6 shows the cereals used to determine the phyto-inhibitory activity of propolis and their characteristics. The temperature of the grains used was 26.5 °C.

### 4.3. Physico–Chemical Analysis

The water activity (a_w_) of propolis was measured at 25 °C using the Aquaspector apparatus AQS-2-TC (Nagy Messsysteme GmbH, Gäufelden, Germany) [52].

Hygroscopicity. The method for determining hygroscopicity proposed by Cai and Corke [53] was used. A total of 10 g of propolis in 100 mL ultra-pure water was centrifuged (70 rpm) in a centrifuge Centra CL2 (Thermo Fisher Scientific Inc., Waltham, MA, USA). The solutions were filtered using Whatman Filter Paper Grade No. 1 (Whatman International Ltd., Maidstone, UK), and the purified propolis extract was frozen at −50° C. The drying of the frozen propolis was performed in a condenser-type freeze dryer (TOPT-12A vacuum freeze dryer, Xi’an, China). A total of 2 g of propolis powder was kept at 25° C in a container with a saturated Na_2_SO_4_ solution (81% RH) for 7 days and then weighed. The hygroscopicity was expressed as g of absorbed water/100 g of propolis.

Water solubility. The Cano-Chauca et al. standard procedure [54] was applied. A total of 2 g of a propolis sample was spun (at 5000 rpm) for 5 min in a Centra CL2 centrifuge (Thermo Fisher Scientific Inc., Waltham, MA, USA) with twenty milliliters of distilled water. A total of 5 mL of the aliquot was dried at 105 °C in an oven. Solubility was measured using the sample’s mass both before and after drying.

Determination of Total Phenolic Content (TPC). The Folin–Ciocalteu reagent technique [55,56] was applied. The TPC was calculated at 760 nm by interpolating propolis absorbance using a calibration curve made with standard gallic acid, 98%. The propolis samples’ total phenolic content was expressed as the gallic acid equivalent (GAE) in one gram of raw propolis. 

Determination of Total Flavonoid Content (TFC). A total of 2.5 mL of 96% ethanol and 1 g of finely ground propolis were centrifuged for 24 h at 200 rpm in a Centra CL2 centrifuge (Thermo Fisher Scientific Inc., Waltham, MA, USA). A 25 mL ethanol 80% adjustment was made to the mixture. A total of 0.1 mL of 10% AlCl_3_, 1.5 mL of 95% ethanol, 0.1 mL of CH_3_COO-K 1M, and 2.8 mL of distilled water were added separately. The mixture was kept in the dark for thirty minutes. The absorbance was measured at 425 nm using the Lambda 20—Perkin Elmer UV/VIS Spectrophotometer (Waltham, MA, USA). The TFC was calculated using a standard quercetin solution and a calibration curve. The TFC was expressed in mg of quercetin equivalents (QE)/100 g of propolis [57].

Ferric Reducing Antioxidant Power (FRAP). The 2,4,6-tripyridyl-S-triazine solution 10 mM (2.5 mL), FeCl_3_·6H_2_O solution 20 mM (2.5 mL), and 25 mL of 300 mM acetate buffer (pH 3.6) were used to prepare the FRAP reagent. In addition, 200 μL of propolis methanolic extracts (1 g in 7 mL methanol) was added along with 1.5 mL of a freshly made FRAP reagent, and the mixture was then maintained at 37 °C for four minutes. After the spectrophotometer calibration with 200 μL of distilled water, the absorbance was measured at 593 nm using the Lambda 20—Perkin Elmer UV/VIS (Waltham, MA, USA). The FeSO_4_ (151.5–9.5 mg/mL) and the FRAP reagent were reacted to create the standard curve. [58,59].

The Antioxidant Activity of Propolis (RSA). In order to prepare the maceration, raw propolis was extracted using a 70% ethanol solution (1:100 *w*/*v*), homogenized continuously for 24 h, and then evaporated until it was completely dry. A mixture of 2,2-diphenyl-1-picrylhydrazyl (DPPH) 0.1 mM ethanoic solution and 0.6 mg/mL propolis solution was produced. The absorbance was measured at 515 nm using the Lambda 20—Perkin Elmer UV/VIS Spectrophotometer (Waltham, MA, USA). At the start of the reaction, as well as after 10 and 20 min, the absorbance (A) was measured. Using DPPH, one can calculate antioxidant activity as follows: % of antioxidant activity (RSA) = (A_DPPH_ − A_sample_)/A_DPPH_ × 100 [60,61].

### 4.4. The Phyto-Inhibitory Activity of Propolis

The phyto-inhibitory effect was determined by assessing the germination duration (inhibiting growth) of specific cereal samples with physico–chemical attributes in standard systems, both with and without the controlled inclusion of propolis [62,63]. In Petri dishes (20 cm^2^) containing a layer of hydrophilic wool, varying amounts of propolis (powder) or aqueous solutions at concentrations of 1%, 5%, and 10% were introduced—this process involved monitoring the selected cereals in the established environment every two days over a 13-day period, with statistical evaluations (averages) conducted on 10 germinated plants.

To produce the aqueous propolis extract, 50 g of propolis was finely chopped and ground into particles, after which 250 mL of distilled water was added, and the mixture was refluxed for 1 h in a round-bottomed flask equipped with a condenser. The resulting heterogeneous mixture underwent centrifugation (~4500× *g*) using a Centra CL2 centrifuge (Thermo Fisher Scientific Inc., Waltham, MA, USA), followed by coarse filtration through a vacuum-connected filter (Merck KgaA, Darmstadt, Germany). Subsequently, another centrifugation at ~4000× *g* took place, and the solution was further filtered (under vacuum) through a low-porosity surface. The final step involved heating at 100 °C until only 20% of the initial quantity remained [64].

### 4.5. Antimicrobial Activity of Propolis

The antibacterial efficacy of aqueous propolis extracts was assessed using three bacterial strains commonly found as contaminants in cereals: *Bacillus subtilis* (ATCC 6633), *Bacillus cereus* (ATCC 11788), and *Proteus mirabilis* (ATCC 7002). Additionally, the antifungal activity was determined against three fungal strains known to contaminate the cereals investigated in our study: *Fusarium oxysporum* (ATCC 48112), *Penicillium chrysogenum* (ATCC 10106), and *Aspergillus niger* (derived from ATCC 16888). All bacterial and fungal strains were procured from MicroBioLogics Inc. (St. Cloud, MN, USA) and Thermo Fisher Scientific Inc. (Waltham, MA, USA). The bacterial and fungal cultures were prepared by diluting overnight cultures in sterile normal saline, and the turbidity of cell suspensions was adjusted to 0.5 McFarland using a McFarland densitometer (Mettler Toledo, Columbus, OH, USA).

The antimicrobial attributes of propolis extracts were examined using the disk diffusion method in accordance with the procedures recommended by CLSI [65]. The diameters of the inhibition zones generated by microorganisms were regarded as a semi-quantitative indicator of the antimicrobial activity. A Mueller–Hinton agar (Merck KgaA, Darmstadt, Germany) was used as the culture medium for bacterial strains, while a Sabouraud 4% dextrose agar (Merck KgaA) was employed for fungal strains. Petri dishes containing culture media were inoculated by flooding with 1 mL of each culture, evenly spreading it across the entire surface.

For the test, 6 mm filter paper discs with 50 μL of each propolis aqueous extract (0.1 g/mL), obtained as described earlier, were utilized. The discs were aseptically positioned on the culture medium surface at approximately equal distances. Discs containing 5 μg ciprofloxacin (Bio-Rad, Marnes-la-Coquette, France) served as a positive control for bacterial growth, while discs with 1 μg voriconazole (Bio-Rad, France) acted as a positive control for fungal growth. Following a 2 h storage at 5 °C, the Petri dishes underwent incubation for 24 h at 37 °C for bacterial growth and 5 days at 25 °C for fungal growth. The measurement of inhibition zone diameters (in mm) was conducted using a DIN 862 ABS digital caliper (Fuzhou Conic Industrial Co., Ltd., Fuzhou, China). All experiments were conducted in triplicate, by the same operator, and under identical laboratory conditions, with the results expressed as the average of the three tests.

### 4.6. Statistical Analysis

Using a two-way analysis of variance (ANOVA), the significance of the variation in inhibition zone diameters was assessed based on the strain type tested and the propolis’s geographic point of origin. In order to examine the relationship between the diameter of the inhibition zone and the flavonoid and phenol content of propolis samples, as well as the germination periods of cereal samples treated with propolis, this study used principal component analysis (PCR) and the Pearson correlation coefficient.

## 5. Conclusions

The bioactive properties of propolis result from the collective action of its various constituents, including flavonoids and phenolic compounds, found in appreciable quantities in propolis from all the regions of Romania. The highest content of phenols and flavonoids was found in propolis samples from Alba and Constanța Counties.

The propolis samples had a phyto-inhibitory effect on the germination of cereal seeds depending on the way they were used (powder or aqueous extract), the concentration used, and the geographical area where they were taken. The propolis samples present antimicrobial activity against all studied bacterial and fungal strains, making them useful in controlling certain plant diseases and infections. 

Due to its properties, Romanian propolis can be integrated with other ecological control methods to manage the infestation of cereal crops. Propolis can be used to manage plant crops in the fight against some microorganisms as an alternative to the intensive, conventional practice in ecological agriculture.

## Figures and Tables

**Table 1:** The values of the physical–chemical parameters for the analyzed propolis samples.

Sample No.	a_w_	Hygroscopicity (g of Absorbed Water/ 100 g Propolis)	Water Solubility (%)	Phenols (mg GAE */g)	Flavonoids (mg QE **/g)	FRAP (mmol Fe^2+^/g)	DPPH (mg GAE */g)
S1	0.71 ± 0.22	14.1 ± 0.9	9.12 ± 0.27	189.4 ± 5.82	84.31 ± 0.09	1.44 ± 0.31	16.44 ± 0.2
S2	0.69 ± 0.15	13.5 ± 0.8	8.98 ± 0.66	172.9 ± 3.25	78.55 ± 0.08	0.98 ± 0.02	15.08 ± 0.2
S3	0.62 ± 0.17	13.1 ± 0.6	8.74 ± 0.50	152.2 ± 6.80	70.10 ± 0.16	1.02 ± 0.06	13.50 ± 0.3
S4	0.74 ± 0.09	12.9 ± 0.9	9.52 ± 0.32	144.0 ± 2.09	81.09 ± 0.98	1.04 ± 0.06	13.92 ± 0.5
S5	0.72 ± 0.18	13.2 ± 0.5	8.26 ± 0.41	138.2 ± 3.06	77.62 ± 0.20	1.15 ± 0.15	12.30 ± 0.4
S6	0.65 ± 0.11	14.7 ± 0.7	11.31 ± 0.58	102.7 ± 2.43	65.30 ± 0.11	0.80 ± 0.09	11.70 ± 0.2
S7	0.78 ± 0.12	12.8 ± 0.6	9.07 ± 0.63	189.0 ± 4.55	85.19 ± 0.07	1.46 ± 0.11	18.30 ± 0.6
S8	0.63 ± 0.16	12.6 ± 0.5	13.43 ± 0.34	126.3 ± 3.14	82.36 ± 0.09	1.22 ± 0.09	15.06 ± 0.4
S9	0.67 ± 0.14	13.3 ± 0.8	12.66 ± 0.75	150.1 ± 4.37	79.54 ± 0.13	1.07 ± 0.07	14.32 ± 0.2

* Gallic acid equivalent. ** Quercetin equivalent.

**Table 2:** The diameters of the inhibition zones (mm) for the nine propolis samples collected from regions of Romania.

Strain	Sample									Ciprofloxacin, 5 μg	Voriconazole, 1 μg
	S1	S2	S3	S4	S5	S6	S7	S8	S9		
*B. subtilis*	28 * ± 1.71	25 * ± 0.00	26 ± 0.57	26 ± 0.57	27 ± 1.14	25 * ± 0.00	26 ± 0.57	26 ± 0.57	25 ± 0.57	30 ± 0.00	-
*B. cereus*	25 ± 1.14	26 ± 1.14	27 ± 1.14	26 ± 0.57	27 ± 0.57	28 * ± 1.71	25 * ± 0.00	26 * ± 0.00	27 * ± 1.71	29 ± 0.00	-
*P. mirabilis*	29 * ± 1.71	24 * ± 0.00	25 * ± 0.00	27 * ± 1.14	28 * ± 1.71	25 * ± 0.00	27 * ± 1.14	25 ± 0.57	24 * ± 0.00	28 ± 0.00	-
*F. oxysporum*	28 * ± 1.14	24 ± 0.57	27 ± 0.57	22 * ± 0.00	26 ± 0.57	23 ± 0.57	25 * ± 0.00	24 * ± 0.00	22 ± 0.57	-	29 ± 0.00
*P. chrysogenum*	22 ± 0.57	17 * ± 0.00	19 * ± 0.57	17 * ± 0.00	18 * ± 0.00	16 * ± 0.00	19 * ± 0.57	17 * ± 0.00	18 * ± 0.57	-	18 ± 0.00
*A. niger*	24 ± 0.57	19 ± 0.57	16 * ± 0.00	16 * ± 0.00	17 ± 0.57	18 * ± 0.00	17 * ± 0.00	18 * ± 0.00	16 * ± 0.00	-	45 ± 0.00

Note: Values are means ± SD of three independent experiments. * The *p*-values of one-way ANOVA indicate no significant differences (*p* > 0.05) among groups.

**Table 3:** Bifactorial variance analysis for propolis samples and different microbial strains.

Dispersion Sums of the Diameters of Inhibition Zones	Quadratic Sum	Degrees of Freedom ν	Variance	F_computed_	F_0.05_
Between the propolis samples	70.15	5	14.03	6.69	2.45
Between strains	732.81	8	91.60	43.70	2.18
Residual, S_r_	83.85	40	2.10	-	-

**Table 4:** Pearson correlation coefficients and the interpretation of correlation for the relation of flavonoid and phenol content of propolis samples with microbial strain type.

Microbial Strains	Flavonoids		Phenols	
	Pearson	Strength and Direction	Pearson	Strength and Direction
*B. subtilis*	0.43	low positive	0.40	low positive
*B. cereus*	−0.88	high negative	−0.82	high negative
*P. mirabilis*	0.43	low positive	0.36	low positive
*F. oxysporum*	0.09	negligible	0.47	low positive
*P. chrysogenum*	0.45	low positive	0.74	high positive
*A. niger*	0.29	negligible	0.42	low positive

**Table 5:** The area, origin, and the landform form where the propolis samples were collected.

Sample	Region	County of Origin	Landforms
S1	Transilvania (Transylvania)	Alba	Mountainous
S2	Crișana	Bihor	Hilly
S3	Maramureș	Maramureș	Mountainous
S4	Banat	Timiș	Plain
S5	Oltenia (Lesser Wallachia)	Golj	Sub-mountainous
S6	Muntenia (Greater Wallachia)	Dâmbovița	Hilly
S7	Dobrogea (Dobruja)	Constanța	Plain
S8	Moldova (Moldavia)	Vaslui	Hilly
S9	Bucovina (Bukovina)	Suceava	Mountainous

**Table 6:** Cereals used and their characteristics.

Cereal Type	Scientific Name	Moisture (%)	Standard Mass Per Storage Volume (kg/hL)
Hexaploid bread wheat	*Triticum aestivum*	13.8	77.1
Maize	*Zea mays* L.	14.4	73.8
Oats	*Avena sativa* L.	12.9	41.1
Barley	*Hordeum vulgare* L.	14.2	63.7

## Data Availability

Data are contained within the article and supplementary materials.

## Supplementary Materials

The following supporting information can be downloaded at https://www.mdpi.com/article/10.3390/antibiotics12121682/s1, Figure S1: Explanation of the total variance of the PCs, respectively the eigenvariance for each of the PCs in the case of the first stage PCA analysis; Figure S2. Explanation of the total variance of the PCs, respectively the eigenvariance for each of the PCs in the case of the second stage PCA analysis; Table S1: Plumule growth lengths (mm) for wheat samples treated with different amounts of propolis powder in time; Table S2: Plumule growth lengths (mm) for wheat samples treated with different concentrations of propolis extract in time; Table S3: Plumule growth lengths (mm) for corn samples treated with different amounts of propolis powder in time; Table S4: Plumule growth lengths (mm) for corn samples treated with different concentrations of propolis extract in time; Table S5: Plumule growth lengths (mm) for barley samples treated with different amounts of propolis powder in time; Table S6: Plumule growth lengths (mm) for barley samples treated with different concentrations of propolis extract in time; Table S7: Plumule growth lengths (mm) for oat samples treated with different amounts of propolis powder in time; Table S8: Plumule growth lengths (mm) for oat samples treated with different concentrations of propolis extract in time.
