# Supplementary material for: Applications of Romanian Propolis in Phyto-Inhibitory Activity and Antimicrobial Protection: A Comparative Study
Source: Antibiotics (Basel). 2023 Nov 30;12(12):1682. doi: 10.3390/antibiotics12121682 (PMC10741215; doi:10.3390/antibiotics12121682)
Supplement: Supplementary file 1 [file antibiotics-12-01682-s001.zip › antibiotics-2715728-supplementary.pdf]

Contents

Phyto-inhibitory activity of propolis samples: **Supplementary Table S1-S8**
Principal Component Analysis: **Supplementary Figure S1-S2**

Supplementary Table S1. Plumule growth lengths (mm) for wheat samples treated with different amounts of propolis powder in time.

Day	1 g									5 g									10 g									M (mm)
	**S1**	**S2**	**S3**	**S4**	**S5**	**S6**	**S7**	**S8**	**S9**	**S1**	**S2**	**S3**	**S4**	**S5**	**S6**	**S7**	**S8**	**S9**	**S1**	**S2**	**S3**	**S4**	**S5**	**S6**	**S7**	**S8**	**S9**	
3	16	16	14	15	15	14	13	15	12	7	5	5	3	4	6	5	6	6	0	0	0	0	0	0	0	0	0	24
5	32	30	31	30	33	31	30	33	29	19	18	15	12	13	16	14	17	16	4	3	4	2	3	2	4	3	2	39
7	80	78	77	77	81	79	76	79	75	55	52	50	51	49	53	54	53	52	18	15	13	10	12	13	14	11	9	82
9	104	98	96	93	107	99	97	100	92	66	64	63	62	60	61	65	61	63	25	24	20	21	22	24	25	23	21	101
11	117	110	113	112	120	116	111	119	109	101	98	96	95	89	90	97	94	98	32	30	25	25	26	28	33	27	26	123
13	138	125	122	120	131	128	125	133	121	113	113	115	111	112	114	116	113	115	41	40	38	36	37	39	42	38	35	145

Supplementary Table S2. Plumule growth lengths (mm) for wheat samples treated with different concentrations of propolis extract in time.

Day	1 %									5 %									10 %									M (mm)
	**S1**	**S2**	**S3**	**S4**	**S5**	**S6**	**S7**	**S8**	**S9**	**S1**	**S2**	**S3**	**S4**	**S5**	**S6**	**S7**	**S8**	**S9**	**S1**	**S2**	**S3**	**S4**	**S5**	**S6**	**S7**	**S8**	**S9**	
3	19	18	16	15	17	16	18	18	14	8	4	5	5	7	5	6	6	4	2	1	1	2	1	2	2	1	0	24
5	36	36	33	35	34	37	34	35	35	24	19	18	16	24	20	21	23	15	11	11	10	8	9	12	7	11	6	39
7	79	80	77	71	78	78	76	79	73	58	53	54	50	59	55	56	57	52	23	22	20	18	19	21	19	21	17	82
9	95	94	90	89	92	94	91	93	90	81	80	75	78	83	79	80	82	74	41	40	38	39	38	39	37	42	35	101
11	115	112	111	113	110	114	116	114	112	102	100	94	96	104	99	103	101	93	52	51	47	48	46	49	50	53	49	123
13	150	165	152	144	160	155	149	163	147	137	132	125	123	141	133	135	138	119	64	65	64	62	63	64	62	64	61	145

Supplementary Table S3. Plumule growth lengths (mm) for corn samples treated with different amounts of propolis powder in time.

**Day**	**1 g**									**5 g**									**10 g**									**M**
	**S1**	**S2**	**S3**	**S4**	**S5**	**S6**	**S7**	**S8**	**S9**	**S1**	**S2**	**S3**	**S4**	**S5**	**S6**	**S7**	**S8**	**S9**	**S1**	**S2**	**S3**	**S4**	**S5**	**S6**	**S7**	**S8**	**S9**	**(mm)**
3	3	2	1	2	1	1	2	1	0	0	0	0	0	0	0	0	0	0	0	0	0	0	0	0	0	0	0	15
5	14	14	14	12	13	14	12	11	11	2	2	1	0	1	1	0	1	0	0	0	0	0	0	0	0	0	0	23
7	28	26	27	24	27	27	24	25	23	21	17	12	10	19	12	16	14	11	4	2	0	1	3	1	2	0	0	37
9	35	33	31	30	33	34	32	30	28	28	24	22	18	27	23	20	21	19	12	10	8	9	11	10	10	8	9	54
11	40	38	40	41	40	39	41	40	37	38	33	32	27	35	37	39	33	28	18	18	15	17	16	15	16	14	13	65
13	53	51	54	52	52	53	50	51	50	49	48	40	34	49	50	48	46	37	23	24	20	21	22	20	21	19	19	83

Supplementary Table S4. Plumule growth lengths (mm) for corn samples treated with different concentrations of propolis extract in time.

**Day**	**1 %**									**5 %**									**10 %**									**M**
	S1	***S2***	***S3***	***S4***	**S5**	**S6**	**S7**	**S8**	**S9**	**S1**	**S2**	**S3**	**S4**	**S5**	**S6**	**S7**	**S8**	**S9**	**S1**	**S2**	**S3**	**S4**	**S5**	**S6**	**S7**	**S8**	**S9**	**(mm)**
3	10	8	9	8	9	8	7	8	7	3	4	2	2	1	2	2	1	1	0	0	0	0	0	0	0	0	0	15
5	21	23	22	18	22	23	20	19	17	6	5	3	4	3	5	4	3	2	4	4	3	4	4	4	3	2	2	23
7	34	34	31	29	33	33	32	30	28	20	18	17	19	18	19	18	17	15	9	10	8	7	10	9	9	7	6	37
9	44	45	40	40	43	44	41	41	39	31	30	31	31	29	30	27	28	26	16	15	14	11	16	14	15	14	13	54
11	53	55	51	53	54	53	52	53	50	42	42	40	39	41	41	40	38	35	20	22	19	17	21	20	19	20	18	65
13	65	64	66	64	64	63	65	62	61	58	60	59	55	57	59	56	54	51	30	31	30	26	30	29	27	26	25	83

Supplementary Table S5. Plumule growth lengths (mm) for barley samples treated with different amounts of propolis powder in time.

**Day**	**1 g**									**5 g**									**10 g**									**M**
	**S1**	**S2**	**S3**	**S4**	**S5**	**S6**	**S7**	**S8**	**S9**	**S1**	**S2**	**S3**	**S4**	**S5**	**S6**	**S7**	**S8**	**S9**	**S1**	**S2**	**S3**	**S4**	**S5**	**S6**	**S7**	**S8**	**S9**	**(mm)**
3	14	12	10	11	13	11	12	11	11	10	10	8	7	11	10	9	8	8	0	0	0	0	0	0	0	0	0	23
5	29	28	29	26	27	26	27	25	26	18	15	17	16	17	16	16	17	14	12	11	10	8	11	11	10	9	9	40
7	38	33	37	34	37	35	39	36	34	29	31	30	27	29	30	28	27	28	20	18	16	19	18	17	19	17	16	55
9	64	66	61	60	65	66	62	64	61	56	55	54	50	53	52	51	51	49	50	52	50	47	52	51	50	47	49	90
11	88	85	83	81	87	83	86	84	82	79	75	77	71	76	74	72	78	66	63	62	56	51	59	60	58	56	53	101
13	110	105	108	107	106	108	105	103	103	91	90	88	89	90	90	87	89	87	80	84	77	73	83	82	80	79	78	132

Supplementary Table S6. Plumule growth lengths (mm) for barley samples treated with different concentrations of propolis extract in time.

Day	1 %									5 %									10 %									M (mm)
	S1	S2	S3	S4	S5	S6	S7	S8	S9	S1	S2	S3	S4	S5	S6	S7	S8	S9	S1	S2	S3	S4	S5	S6	S7	S8	S9	
3	19	17	16	15	18	15	16	16	14	12	9	11	8	11	11	10	9	9	2	1	1	1	1	1	1	0	0	23
5	34	35	33	29	35	34	32	30	31	23	20	21	23	22	22	20	21	19	17	15	18	14	18	17	15	17	16	40
7	45	42	46	40	44	43	41	42	43	31	37	34	30	36	35	33	32	29	24	22	23	20	26	25	22	24	24	55
9	70	67	69	66	71	68	67	65	68	44	42	44	45	45	44	42	43	41	52	51	50	47	48	50	49	51	39	90
11	92	90	91	89	90	88	89	86	85	82	81	89	82	88	87	83	77	78	69	67	71	59	67	66	70	69	60	101
13	107	101	113	97	112	109	106	102	98	96	98	95	92	97	96	96	94	93	83	82	88	79	85	86	83	81	80	132

Supplementary Table S7. Plumule growth lengths (mm) for oat samples treated with different amounts of propolis powder in time.

Day	1 g									5 g									10 g									M (mm)
	S1	S2	S3	S4	S5	S6	S7	S8	S9	S1	S2	S3	S4	S5	S6	S7	S8	S9	S1	S2	S3	S4	S5	S6	S7	S8	S9	
3	16	15	18	19	15	17	16	16	14	5	5	4	5	4	4	5	4	3	0	0	0	0	0	0	0	0	0	25
5	24	22	24	24	23	25	23	22	22	11	12	10	11	10	12	11	11	9	4	2	2	1	3	2	2	1	1	30
7	33	32	33	32	32	31	33	32	30	21	22	20	22	21	21	22	21	20	12	11	9	8	11	10	9	8	7	44
9	42	41	40	42	41	40	41	40	38	37	39	32	38	36	37	35	34	31	23	22	22	21	22	21	20	21	20	65
11	51	55	56	52	52	54	53	53	50	45	44	48	42	47	46	43	41	42	30	27	28	23	30	27	29	26	25	102
13	60	62	63	59	61	62	61	60	59	58	50	53	52	53	52	51	50	50	39	36	33	38	38	36	36	37	34	123

Supplementary Table S8. Plumule growth lengths (mm) for oat samples treated with different concentrations of propolis extract in time.

Day	1 %									5 %									10 %									M (mm)
	S1	S2	S3	S4	S5	S6	S7	S8	S9	S1	S2	S3	S4	S5	S6	S7	S8	S9	S1	S2	S3	S4	S5	S6	S7	S8	S9	
3	21	19	17	18	20	19	21	20	16	9	9	8	7	10	8	8	9	6	1	0	0	0	1	0	0	1	0	25
5	30	27	28	23	31	26	29	30	22	18	17	19	18	16	19	18	16	14	10	9	7	8	6	4	4	5	4	30
7	43	42	44	40	44	40	43	41	39	28	27	23	26	25	26	26	24	22	17	18	15	13	16	12	14	17	11	44
9	50	48	49	47	52	51	49	50	46	42	43	41	45	44	42	41	40	39	28	25	22	27	29	24	25	26	21	65
11	70	71	70	68	69	67	59	64	58	51	54	50	51	53	52	51	52	50	45	44	43	40	44	40	41	43	39	102
13	81	80	82	75	81	79	80	79	76	62	66	60	63	65	62	60	64	59	53	55	52	50	54	51	53	54	49	123

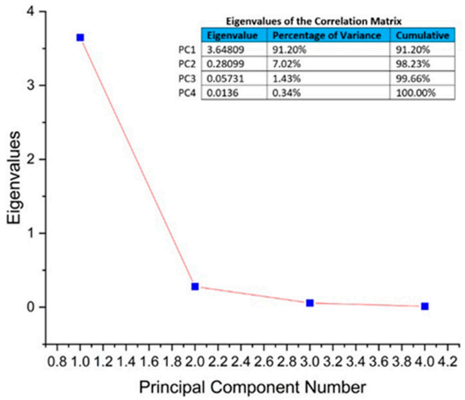

	Eigenvalue	Percentage of Variance	Cumulative
PC1	3.64809	91.20%	91.20%
PC2	0.28099	7.02%	98.23%
PC3	0.05731	1.43%	99.66%
PC4	0.0136	0.34%	100.00%

Supplementary Figure S1. Explanation of the total variance of the PCs, respectively the eigenvariance for each of the PCs in the case of the first stage PCA analysis.

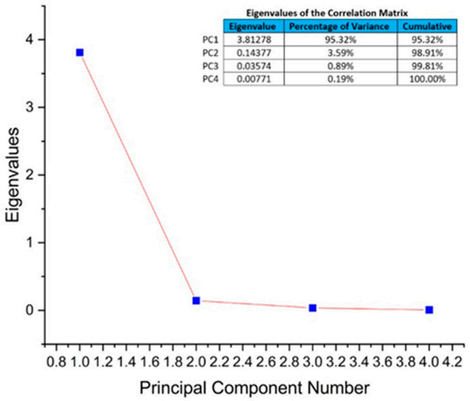

	Eigenvalue	Percentage of Variance	Cumulative
PC1	3.81278	95.32%	95.32%
PC2	0.14377	3.59%	98.91%
PC3	0.03574	0.89%	99.81%
PC4	0.00771	0.19%	100.00%

Supplementary Figure S2. Explanation of the total variance of the PCs, respectively the eigenvariance for each of the PCs in the case of the second stage PCA analysis.
